# Clinical characteristics and outcomes of patients with acute myelogenous leukemia admitted to intensive care: a case-control study

**DOI:** 10.1186/1471-2407-10-516

**Published:** 2010-09-28

**Authors:** Amanda L Roze des Ordons, Kris Chan, Imran Mirza, Derek R Townsend, Sean M Bagshaw

**Affiliations:** 1 Department of Anesthesia and Pain Medicine, Faculty of Medicine and Dentistry, University of Alberta Hospital, 8440-112ST NW, Edmonton, Alberta, T6G2B7 Canada; 2 Department of Medicine, Faculty of Medicine and Dentistry, University of Alberta Hospital, 8440-112ST NW, Edmonton, Alberta, T6G2B7 Canada; 3 Department of Laboratory Medicine and Pathology, Faculty of Medicine and Dentistry, University of Alberta Hospital, 8440-112ST NW, Edmonton, Alberta, T6G2B7 Canada; 4 Division of Critical Care Medicine, Faculty of Medicine and Dentistry, University of Alberta Hospital, 8440-112ST NW, Edmonton, Alberta, T6G2B7 Canada

## Abstract

**Background:**

There is limited epidemiologic data on patients with acute myelogenous (myeloid) leukemia (AML) requiring life-sustaining therapies in the intensive care unit (ICU). Our objectives were to describe the clinical characteristics and outcomes in critically ill AML patients.

**Methods:**

This was a retrospective case-control study. Cases were defined as adult patients with a primary diagnosis of AML admitted to ICU at the University of Alberta Hospital between January 1^st ^2002 and June 30^th ^2008. Each case was matched by age, sex, and illness severity (ICU only) to two control groups: hospitalized AML controls, and non-AML ICU controls. Data were extracted on demographics, course of hospitalization, and clinical outcomes.

**Results:**

In total, 45 AML patients with available data were admitted to ICU. Mean (SD) age was 54.8 (13.1) years and 28.9% were female. Primary diagnoses were sepsis (32.6%) and respiratory failure (37.3%). Mean (SD) APACHE II score was 30.3 (10.3), SOFA score 12.6 (4.0) with 62.2% receiving mechanical ventilation, 55.6% vasoactive therapy, and 26.7% renal replacement therapy. Crude in-hospital, 90-day and 1-year mortality was 44.4%, 51.1% and 71.1%, respectively. AML cases had significantly higher adjusted-hazards of death (HR 2.23; 95% CI, 1.38-3.60, p = 0.001) compared to both non-AML ICU controls (HR 1.69; 95% CI, 1.11-2.58, p = 0.02) and hospitalized AML controls (OR 1.0, reference variable). Factors associated with ICU mortality by univariate analysis included older age, AML subtype, higher baseline SOFA score, no change or an increase in early SOFA score, shock, vasoactive therapy and mechanical ventilation. Active chemotherapy in ICU was associated with lower mortality.

**Conclusions:**

AML patients may represent a minority of all critically ill admissions; however, are not uncommonly supported in ICU. These AML patients are characterized by high illness severity, multi-organ dysfunction, and high treatment intensity and have a higher risk of death when compared with matched hospitalized AML or non-AML ICU controls. The absence of early improvement in organ failure may be a useful predictor for mortality for AML patients admitted to ICU.

## Background

Acute myelogenous (myeloid) leukemia (AML), a hematologic malignancy characterized by clonal proliferation of myeloblasts, is the most common type of leukemia in adults and uniformly fatal without treatment[1]. Over the past 20 years, more aggressive chemotherapeutic regimens, availability of bone marrow transplantation (BMT), and development of subtype-directed therapies have led to improved survival[2]. However, these more aggressive therapeutic strategies have also contributed to a higher likelihood of complications [3,4], translating into a greater demand for support in an intensive care unit (ICU) setting[5-10]. Moreover, ICU support of these patients has been associated with more intensive and greater resource utilization[11].

A number of observational studies in patients with hematologic malignancies admitted to ICU have described the clinical outcomes and prognostic factors for survival [5-8,11-22]. The majority of studies have been small, single centre, retrospective cases-series and have enrolled a mixed cohort of critically ill patients with all forms of hematologic malignancy or who had received a BMT.(Additional File 1) Few studies have specifically investigated the ICU course in AML patients[13,14,16,17]. Two primarily focused on AML patients with pulmonary infiltrates requiring mechanical ventilation, and reported a dismal ICU survival of 3-13%[14,17]. In another study of 83 consecutive AML patients, Rabbat et al reported 66% ICU survival and 33% 1-year survival[13]. In a cohort of 90 consecutive patients admitted to ICU over 3-year surveillance, of which 82% had a primary AML diagnosis, Thakkar et al described poor 6 and 12 month survival of only 18% and 16%, respectively[16]. Finally, Park et al described 68% hospital mortality in 50 acute leukemia patients, 70% with AML, admitted to ICU with septic shock[16]. However, clear inferences regarding prognosis for critically ill AML patients from these studies are limited due to selection bias (i.e. only receiving mechanical ventilation), inclusion of a mixed leukemia population (i.e. acute lymphoblastic leukemia), or lack of controls for comparison.

Accordingly, we performed a retrospective case-control study of patients with AML admitted to ICU. We hypothesized that AML patients receiving ICU support would have higher mortality when compared with matched controls; however, that a considerable proportion of these AML patients would survive to leave hospital. Our objectives were to: 1) define the incidence of ICU admissions with a primary diagnosis of AML; 2) describe the clinical characteristics, course and treatment intensity of ICU admissions with AML; and 3) describe the short- and long-term outcomes of ICU AML patients compared with matched non-AML ICU patients and non-ICU AML hospitalized patients.

## Methods

### Study Design, Setting and Population

We performed a retrospective case-control study of patients with acute myelogenous (myeloid) leukemia (AML) admitted to the General Systems Intensive Care Unit (GSICU) at the University of Alberta Hospital between January 1^st^, 2002 and June 30^th^, 2008. Cases were defined as adult patients (age ≥18 years) with a confirmed diagnosis of AML and admission to the GSICU. Each case was then matched with two control groups: 1) non-ICU hospitalized patients with confirmed AML (i.e. admitted to the medical/hematology ward; matched 1:1); and subsequently to 2) non-AML critically ill patients (i.e. admitted to the GSICU; matched 1:5). The study protocol was approved by the Health Research Ethics Board at the University of Alberta prior to commencement.

### Study Definitions

AML diagnosis was typed and characterized according to the WHO and FAB classification schemes[23]. The status of AML was categorized as: newly diagnosed/active, refractory or relapse, in remission following HSCT, or in remission. AML patients were also classified according to treatment regimen received: intensive induction/consolidative chemotherapy, or cytoreductive chemotherapy and/or palliation. Patients receiving induction/consolidative chemotherapy for AML at our institution do not routinely receive prophylactic antibiotic or antifungal therapy. Neutropenia was defined as an absolute neutrophil count < 1.0 × 10^9 ^cells/L. When patients develop an episode of febrile neutropenia (i.e. documented temperature ≥38.3°C and ANC ≤1.0 cells/10^9^) empiric broad-spectrum antimicrobials are initiated. The presence of co-morbid illness was determined and quantified. Non-AML leukemia, lymphoma or solid organ tumor were defined by confirmatory pathologic investigations. Congestive heart failure (CHF) was defined as the presence of New York Heart Association (NYHA) class III or IV disease. Chronic lung disease was defined as functional limitation or home oxygen therapy attributable to documented lung pathology. Chronic kidney disease (CKD) was defined as National Kidney Foundation Kidney Disease Outcome Quality Initiative (KDIGO) ≥ stage 4 disease. Liver disease was defined as biopsy proven cirrhosis or elevated liver enzymes attributable to active liver disease. Human immunodeficiency virus (HIV) or acquired immunodeficiency syndrome (AIDS) were defined by documented HIV status and/or documented opportunistic infection/illness attributable to HIV. Shock was defined by a documented mean arterial pressure (MAP) ≤60 mmHg and/or use of vasoactive therapy and an elevated serum lactate. Illness severity was defined according to the Acute Physiology and Chronic Health Evaluation (APACHE) II score[24]. Baseline and changes to organ failure were characterized by the Sequential Organ Failure Assessment (SOFA) score[25]. Acute kidney injury (AKI) was defined as an acute rise in serum creatinine ≥150 μmol/L or urine output ≤400 mL/24 hr and no documented CKD (≥ stage 4).

### Study Protocol

Cases were identified by review of a local database of consecutive new diagnoses of AML maintained by the Department of Laboratory Medicine at the University of Alberta Hospital. This database maintains detailed data on the clinical and diagnostic characteristics of AML patients (i.e. WHO classification, cytogenetic profile, laboratory values at diagnosis). These AML patients were subsequently cross referenced with an ICU-specific database, the Minimal Data Set (MDS) database, which routinely captures demographic, clinical, physiologic and outcome data on all GSICU admissions. The linkage of these two datasets enabled capture of "cases" of confirmed AML and GSICU admission. Each case was subsequently matched to the two control groups. Non-ICU hospitalized AML controls were randomly selected from the AML database and matched for age (± 2 years) and sex. Non-AML ICU controls were randomly selected from the MDS dataset, and matched for age (± 2 years), sex and APACHE II score (± 2 points) (ICU cohort only).

Detailed data pertaining to demographics, clinical characteristics, acute physiology, course in hospital, and clinical outcomes were extracted from the medical records of each patient and merged with data obtained from the AML and MDS databases. Demographic and baseline clinical data included age, sex, co-morbid disease, primary diagnosis, surgical status, and dates of hospital/ICU admission and discharge. The characteristics of AML were extracted from the AML database. For cases and ICU controls, acute physiologic data, laboratory parameters, and clinical course, interventions, including corticosteroids, AML-specific chemotherapy, vasoactive therapy, mechanical ventilation and renal replacement therapy (RRT) were recorded. SOFA scores were ascertained at baseline and at days 1, 3 and 7 after ICU admission.

The primary outcome of interest was the incidence of ICU admissions for patients with a diagnosis of AML, defined as a proportion (number of cases per number of individual admissions) and as number of cases per 1000 ICU admissions. For patients with more than one ICU admission within the same hospitalization, only the first ICU episode was considered. Secondary outcomes of interest included short-term (ICU/hospital) and long-term (90-day, 1-year) survival, ICU/hospital lengths of stay, changes in organ failure scores during the first week in ICU (i.e. ΔSOFA), and measures of treatment intensity (i.e. need for mechanical ventilation, vasoactive therapy, and RRT). Tertiary outcomes included determination of factors associated with survival to discharge from ICU.

### Statistical Analysis

Clinical variables and univariate comparison between groups are reported as means with standard deviations (SD) for normally or near normally distributed variables and compared using Student's t-test or analysis of variance (ANOVA); non-normally distributed continuous data is reported as medians with inter-quartile ranges (IQR) and compared using Mann Whitney U test or Kruskal Wallis test, as appropriate. Categorical data are reported as proportions and compared using Fisher's Exact Test. Crude survival stratified by study group was assessed graphically by the Kaplan-Meier product limit estimator and compared with the log-rank test. Cox proportional hazards analysis was used to evaluate the association between AML cases and controls and mortality. Data are presented as crude and covariate-adjusted hazard ratios (HR) and 95% confidence intervals (CI). Plots of log (-log [survival]) versus log (survival time) were constructed to evaluate the assumption of proportionality. Univariate analysis of AML cases was performed to evaluate for clinical factors associated with ICU survival. Intercooled Stata Release 10.2 (Stata Corp, TX) was used for all data analysis. A p-value of <0.05 was considered statistically significant for all comparisons.

## Results

The baseline demographics and clinical features of patients stratified by group are shown in Table 1. There were 386 patients diagnosed with AML during the study period. A total of 50 AML patients, representing 13% of all new AML diagnoses, were admitted to ICU. This represented an estimated incidence of 0.74% of all ICU admissions (n = 6,801) or 7.4 AML patients per 1000 ICU admissions. The medical records for five of these AML ICU patients could not be retrieved for detailed review; therefore, these patients were omitted from further analysis.

**Table 1:** Summary of baseline demographic and clinical features of patients stratified by group.

Variable	Total (n = 320)	AML ICU Cases (n = 45)	AML non-ICU Controls (n = 50)	Non-AML ICU Controls (n = 225)	p-value
**Age (mean [SD]) (years)**	54.6 (13.7)	54.8 (13.1)	53.4 (16.2)	54.8 (13.2)	0.75

**Female sex (%)**	91 (28.4)	13 (28.9)	11 (22.0)	67 (29.8)	0.56

**BMI (mean [SD]) (kg/m^2^)**	29.3 (7.1)	30.7 (6.6)	29.8 (6.4)	27.0 (7.9)	0.13

**Co-morbid disease type (%)**					
**Leukemia**	102 (31.9)	45 (100)	50 (100)	7 (3.1)	< 0.001
**Lymphoma**	5 (1.6)	2 (2.2)	0 (0)	4 (1.8)	0.81
**Solid organ tumor**	8 (2.5)	1 (2.2)	0 (0)	7 (3.1)	0.64
**Congestive heart failure**	11 (3.4)	1 (2.2)	3 (6.0)	7 (3.1)	0.62
**Chronic lung Disease**	16 (5.0)	0 (0)	3 (6.0)	13 (5.8)	0.28
**Chronic kidney disease**	19 (5.9)	0 (0)	2 (4.0)	17 (7.6)	0.13
**Liver disease**	41 (12.8)	1 (2.2)	2 (4.0)	38 (16.9)	0.002
**HIV/AIDS**	5 (1.6)	0 (0)	1 (2.0)	4 (1.8)	1.0

**Co-morbid disease (%)^¶^**					
None (%)	143 (44.7)	24 (53.3)	0 (0)	119 (52.9)	< 0.001
1	149 (46.6)	19 (42.2)	41 (82.0)	89 (39.6)	
2	26 (8.1)	2 (4.4)	7 (14.0)	17 (7.6)	
≥3	2 (0.6)	0 (0)	2 (4.0)	0 (0)	

**Surgical admission (%)**	54 (16.9)	1 (2.2)	0 (0)	53 (23.6)	< 0.001

Abbreviations: BMI = body mass index; HIV = human immunodeficiency virus; AIDS = acquired immunodeficiency syndrome

§ Included prior myocardial infarction, congestive heart failure, and/or peripheral vascular disease

¶ Co-morbid disease excluding AML diagnosis

### Demographic, Clinical and Diagnostic Characteristics of AML

AML cases had a mean (SD) age of 54.8 (13.1) years, 28.9% were female, and had a median (IQR) one (0-2) co-morbid illness (Table 1). There were no statistical differences in the AML classification, cytogenetic profile or therapeutic regimen between AML cases and controls (Table 2). There were more AML cases classified as remission when compared with AML controls. Of these, four had received HSCT after intensive chemotherapy. The median (IQR) duration from AML diagnosis to ICU admission was 46 days (12-251). This duration was shortest in newly diagnosed or those with active disease (17 days [2-28]) and in those in remission post-HSCT (93 [88-964]) compared to those with refractory/relapsed disease (323 [144-743]) or remission following chemotherapy alone (280 [102-436]) (p = 0.0001).

**Table 2:** Summary of diagnostic characteristics of AML.

Variable	Total (n = 95)	AML ICU Cases (n = 45)	AML non-ICU Controls (n = 50)	p-value
**AML WHO classification (n = 91)**				
AML with recurrent genetic abnormalities	44 (46.3)	19 (42.2)	25 (50.0)	0.18
AML with multi-lineage dysplasia	17 (17.9)	10 (22.2)	7 (14.0)	
AML and myelodysplastic syndromes, therapy-related	6 (6.3)	5 (11.1)	1 (2.0)	
AML not otherwise categorized	28 (29.5)	11 (24.4)	17 (34.0)	

**AML FAB Classification (n = 71)**				
M0	4 (4.2)	0 (0)	4 (8.0)	0.12
M1	28 (29.5)	15 (33.3)	13 (26.0)	
M2	20 (21.1)	11 (24.4)	9 (18.0)	
M3	12 (12.6)	3 (6.7)	9 (18.0)	
M4	12 (12.6)	4 (8.9)	8 (16.0)	
M4eo	5 (5.3)	4 (8.9)	1 (2.0)	
M5	13 (13.7)	7 (15.6)	6 (12.0)	
M6	1 (1.1)	1 (2.2)	0 (0)	

**Cytogenetic prognosis^§ ^(%)**				
Good	13 (13.7)	6 (13.3)	7 (14.0)	0.96
Intermediate	38 (40.0)	17 (37.8)	21 (42.0)	
Poor	14 (28.0)	13 (28.9)	14 (28.0)	
Unknown/not available	8 (16.0)	9 (20.0)	8 (16.0)	

**Complete blood count at diagnosis:**				
Hemoglobin (g/L)	96.7 (22.1)	95.9 (21.6)	97.3 22.6)	0.76
Platelets (cells/10^9^)	46 (26-96)	45 (31-96)	47 (24-96)	0.46

**White cell count (WBC) (cells/10^9^)**	8.2 (2.3-50.6)	7.7 (2.0-50.8)	16.0 (3.2-42.9)	0.87
WBC < 1 (%)	6 (7.1)	3 (6.7)	3 (6.0)	1.0
WBC > 25 (%)	34 (35.8)	15 (33.3)	19 (38.0)	0.67

**Duration of Neutropenia (days) (mean [SD])**	14.1 (14.3)	13.1 (14.4)	15.2 (14.2)	0.48

**Bone marrow blast count (%)**	56.6 (23.7)	52.9 (23.7)	60.1 (23.5)	0.15
Blasts >20%	86 (94.5)	41 (93.2)	45 (95.7)	0.67

**AML Status**				
Newly diagnosed/active disease	58 (61.1)	24 (53.3)	34 (68.0)	0.01
Refractory/relapse	29 (30.5)	13 (28.9)	16 (32.0)	
BMT/remission	4 (4.2)	4 (8.9)	0 (0)	
Remission	4 (4.2)	4 (8.9)	0 (0)	

**Therapeutic Regimen**				
Intensive/induction chemotherapy	78 (82.1)	38 (84.4)	40 (80.0)	0.60
Cytoreductive/palliative	17 (17.9)	7 (15.6)	10 (20.0)	

Abbreviations: WHO = World Health Organization; FAB = French American British; WBC = white blood cell count

§ Cytogenetic Prognosis: *Good *= t(8;21); t(15;17); inv16 or t(16;16); *Intermediate *= normal; trisomy 8; 11q23 abnormality; *Poor *= deletion of chromosome 5 or 5q; deletion of chromosome 7 or 7q; 3q abnormality; complex; *Unknown *= significance not known; no cytogenetic information.

### Baseline Characteristics, Physiology and Laboratory Parameters in ICU

AML cases, when compared to ICU controls, had a higher rate of admission for sepsis and a non-significant but higher rate for respiratory failure (Table 3). Only one AML case (2.2%) was post-operative, compared with 23.6% of ICU controls (p < 0.001). As expected, there was no significant difference in mean (SD) APACHE II scores at ICU admission (p = 0.39) (Table 3). AML cases had a lower GCS score (p = 0.0003), higher temperature (p = 0.05), higher heart rate p = 0.001), and respiratory rate (p < 0.0001) when compared with ICU controls.

**Table 3:** Summary of ICU admission characteristics, acute physiology, and laboratory parameters stratified by AML status.

Variable	Total (n = 275)	AML ICU Cases (n = 45)	Non-AML ICU Controls (n = 225)	p-value
**Admission diagnostic category (%)^§^**				

Sepsis/Infectious	53 (19.8)	14 (32.6)	39 (17.3)	0.04

Respiratory	70 (26.1)	16 (37.2)	54 (24.0)	0.09

Genitourinary	7 (2.6)	0 (0)	7 (3.1)	0.60

Gastrointestinal	50 (18.7)	1 (2.3)	49 (21.8)	0.001

Cardiovascular	27 (10.1)	1 (2.3)	26 (11.6)	0.09

Neurologic	15 (5.6)	2 (4.7)	13 (5.8)	1.0

Endocrine/Metabolic	13 (4.9)	0 (0)	13 (5.8)	0.14

Trauma	22 (8.2)	0 (0)	22 (9.8)	0.03

Other	11 (4.1)	9 (20.9)	2 (0.9)	< 0.001

APACHE II score (mean [SD])	29.1 (9.9)	30.3 (10.3)	28.9 (9.9)	0.39

APACHE II score > 25 (%)	185 (68.5)	35 (77.8)	150 (66.7)	0.16

SOFA score (mean [SD])	11.7 (4.6)	12.6 (4.0)	10.8 (4.9)	0.05

SOFA score > 11 (%)	55 (57.9)	32 (71.1)	23 (46.0)	0.02

Mechanical ventilation (%)	225 (83.3)	30 (66.7)	195 (86.7)	0.003

**Acute physiology**				

GCS (mean [SD])	7.6 (3.4)	5.9 (3.4)	7.9 (3.3)	0.0003

Temperature_max _(mean [SD]) (degrees C)	37.4 (3.6)	38.4 (1.2)	37.2 (3.8)	0.05

Heart rate_max _(mean [SD]) (/min)	118 (23.4)	128 (18)	116 (24)	0.001

Respiratory rate_max _(mean [SD]) (/min)	28 (8)	34 (8)	26 (8)	< 0.0001

Mean arterial pressure_min _(mean [SD]) (mmHg)	59 (17)	60 (17)	59 (17)	0.74

**Laboratory parameters**				

Hematocrit (mean [SD])	26.6 (6.7)	22.6 (5.0)	27.3 (6.7)	< 0.0001

White blood cell count (med [IQR]) (10^9 ^cells/mL)	7.8 (4.2-13.1)	0.6 (0.2-7.5)	9.1 (4.8-13.6)	< 0.0001

Bilirubin (med [IQR]) (μmol/L)	23 (14-49)	33 (19-48)	22 (13-52)	0.26

PaO_2_/FiO_2 _ratio (mean [SD])	174 (97)	158 (85)	177 (99)	0.22

pH (mean [SD])	7.25 (0.15)	7.30 (0.13)	7.24 (0.15)	0.03

Serum Sodium (mean [SD]) (mmol/L)	137 (6.0)	137 (7.0)	137 (5.8)	0.63

Serum Creatinine (med [IQR) (μmol/L)	168 (93-284)	145 (97-203)	170 (89-318)	0.13

Urine output (med [IQR]) (L/24 hr)	1.3 (0.4-2.5)	2.2 (1.0-3.1)	1.2 (0.4-2.4)	0.0006
Urine output <400 mL (%)	61 (22.9)	3 (7.0)	58 (25.9)	0.005

Acute kidney injury (%)	138 (52.7)	23 (53.5)	115 (52.5)	1.0

Abbreviations: SOFA = sequential organ failure assessment; APACHE = acute physiology and chronic health evaluation; GCS = Glasgow coma score; ANC = absolute neutrophil count

§ Sum exceeds 100% due to more than one primary diagnosis.

### Treatment Intensity and Course in ICU for AML patients

AML cases were significantly less likely to receive mechanical ventilation (p = 0.003) despite comparable or low PaO_2_/FiO_2 _ratios. Of AML cases, 88.4% presented with shock, and 75.6% were supported with vasoactive therapy. Acute kidney injury occurred in 53.5% with 47.8% of these patients subsequently receiving support with acute RRT. In total, 97.8% received broad-spectrum antimicrobials, 86.7% received transfused blood products, 48.9% received replacement corticosteroids, 51.2% received G-CSF and 15.6% received AML-specific chemotherapy while in ICU. At the time of ICU admission, 93.3% were designated as full resuscitation, however, 47.6% later had their status changed to not for resuscitation (NFR) while in ICU. Of the 17 ICU deaths in AML patients, 88.2% occurred in those with a change in NFR status (p < 0.0001 compared with ICU mortality for no change in NFR status).

### Survival, Lengths of Stay and Discharge Disposition

**Figure 1:**
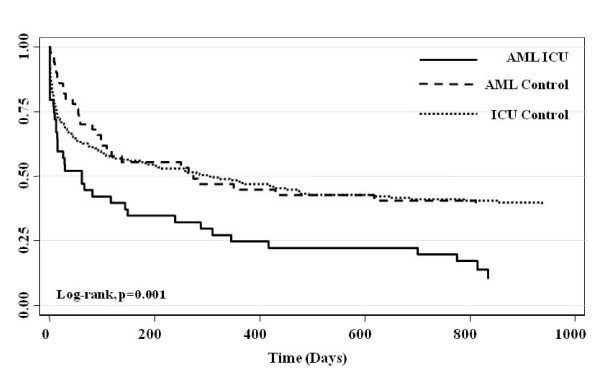
**Crude K-M survival estimates by group (truncated at 1000 days)**.

**Figure 2:**
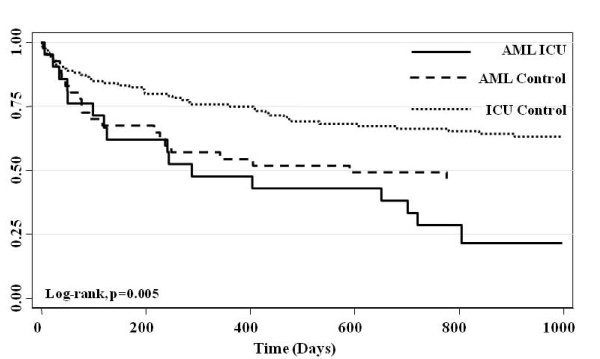
**Crude K-M survival estimates by group conditional on survival to hospital discharge (truncated at 1000 days)**.

Crude survival at hospital discharge, 28-days, 90-days and 1-year was lower for AML cases compared with both AML and ICU controls (Table 4, Figure 1). At 1-year, survival among AML cases was 28.9%, compared with 52.0% and 54.0% for AML and ICU controls, respectively (p = 0.03). The median survival for AML cases admitted to ICU was 82 days (95% CI, 14-700), and was significantly lower than for AML and ICU controls (Table 4). By Cox survival analysis, AML cases had a significantly higher adjusted-hazards ratio (HR) for death (HR 2.23; 95% CI, 1.38-3.60) compared with both ICU (HR 1.69; 95% CI 1.11-2.58) and AML controls (HR 1.0, reference) (Table 5). ICU and hospital stay were non-significantly longer for AML cases surviving to ICU and hospital discharge (Table 5). In total, 46.7% of AML cases survived hospitalization and were discharged home compared with 40.9% of ICU controls (p = 0.32) and 62.0% of AML controls (p = 0.15). By Cox survival analysis, the adjusted-HR of death, conditional on survival to hospital discharge, was significantly higher for AML cases (HR 2.46; 95% CI, 1.4-4.3, p = 0.002) compared to either AML controls (HR 1.40; 95% CI, 0.85-2.30, p = 0.19) or ICU controls (HR 1.0, reference).(Figure 2)

**Table 4:** Summary of clinical outcomes stratified by group.

Outcome	Total (n = 145)	AML ICU Cases (n = 45)	AML non-ICU Controls (n = 50)	Non-AML ICU Controls (n = 225)	p-value
**ICU death (%)**	83 (30.7)	17 (37.8)	-	66 (29.3)	0.29

**Hospital death (%)**	121 (37.8)	20 (44.4)	9 (18.0)	92 (40.9)	0.004

**28-Day death (%)**	98 (30.6)	17 (37.8)	8 (16.0)	73 (32.4)	0.03

**90-Day death (%)**	132 (41.3)	23 (51.1)	16 (32.0)	93 (41.3)	0.17

**1-Year death (%)**	178 (55.6)	32 (71.1)	27 (54.0)	119 (52.9)	0.08

**ICU length of stay (days)**	4 (2-11)	5 (2-12)	-	4 (2-11)	0.38

Survived	5 (2-12)	8 (2-14)	-	5 (3-11)	0.44
Dead	2 (1-5)	4 (1-7)	-	1 (1-3)	0.17

**Hospital length of stay (days)**	15 (6-39)	22 (11-47)	24 (9-35)	13 (5-36)	0.12
Survived	21 (10-45)	37 (18-50)	25 (12-35)	17 (10-44)	0.22
Dead	14 (9-28)	13 (9-19)	6 (1-28)	3 (1-12)	0.17

**Median survival (95% CI) (days)**	207 (14-2442)	82 (14-700)	274 (55-1303)	258 (9-2442)	0.01

**Table 5:** Cox proportional hazards survival analysis stratified by group.

Group	Crude HR (95% CI)	Adjusted^§ ^HR (95% CI)
**AML controls**	1.0^¶^	1.0^¶^

**ICU controls**	1.24 (0.84-1.83)	1.69 (1.11-2.58)

**AML cases**	2.12 (1.31-3.43)	2.23 (1.38-3.60)

Abbreviations: AML = acute myelogenous leukemia; ICU = intensive care unit; CI = confidence interval

§ Cox PH model adjusted for: age, sex, co-morbid disease, surgical status.

¶ Reference variable

### Prognostic Factors for AML Patients in ICU

**Figure 3:**
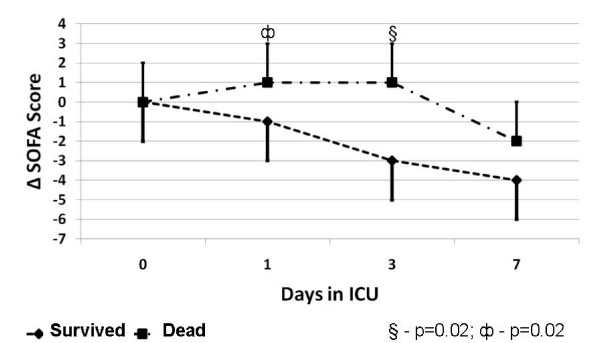
**Δ SOFA score over first 7 days in ICU stratified by 90-day survival**.

For AML cases, several factors were found to be associated with ICU mortality by univariate analysis (Table 6). Older age, AML subtype M1, higher organ failure score, shock, vasoactive therapy, mechanical ventilation were all associated with lower likelihood of survival. Alternatively, AML patients receiving AML-specific chemotherapy while in ICU had higher survival. The absence of change or worsening in organ failure score in the first three days after ICU admission was also associated with lower survival (Figure 3). In this cohort, no significant association was found between 90-day survival and co-morbid illness, cytogenetic profile, time from AML diagnosis to ICU admission, relapse or refractory AML status, primary septic diagnosis, or RRT.

**Table 6:** Univariate factors associated with 90-day mortality in patients with AML.

Characteristic	90-Day Status		p-value
		
	Dead (n = 25)	Alive (n = 20)	
**Age (mean [SD])**	59.4 (11.7)	49.8 (12.9)	0.01

**≥ 2 co-morbidities (%)**	12 (52.2)	9 (40.9)	0.55

**AML M1 Subtype (%)**	12 (48.0)	3 (15.0)	0.03

**Poor cytogenetic profile (%)**	6 (26.1)	7 (31.8)	0.75

**Time from AML diagnosis to ICU (d)**	65 (12-288)	40 (9-95)	0.61

**Relapse/refractory AML status (%)**	8 (32.0)	5 (25.0)	0.75

**Duration of neutropenia (d) (med [IQR])**	15 (13-19)	15 (13-19)	0.80

**Chemotherapy in ICU (%)**	1 (4.0)	6 (30.0)	0.03

**Sepsis diagnosis (%)**	8 (32.0)	6 (33.3)	1.0

**APACHE II score (mean [SD])**	32.4 (10.7)	28.0 (9.5)	0.15

**SOFA score (mean [SD])**	14.6 (4.3)	11.4 (3.3)	0.007

**Delta SOFA (day 1) (med [IQR])**	1 (0 to 3)	0 (-2 to 2)	0.02

**Delta SOFA (day 3) (med [IQR])**	2 (-1 to 4)	-2 (-4 to 2)	0.02

**SOFA (maximum) (mean [SD])**	12.1 (5.3)	10.8 (3.1)	0.46

**MAP <60 mmHg (%)**	11 (44.0)	4 (22.2)	0.20

**Lactate (mmol/L) (mean [SD])**	4.1 (4.6)	2.1 (1.7)	0.08

**Vasoactive therapy (%)**	22 (88.0)	12 (60.0)	0.04

**Shock (%)**	25 (100)	13 (72.2)	0.009

**Platelets (10^9 ^cells/L) (med [IQR])**	17 (9-37)	14 (10-31)	0.90

**Bilirubin (μmol/L) (mean [SD])**	40.5 (37.3)	31.1 (15.7)	0.30

**P/F ratio (mean [SD])**	116 (88)	149 (100)	< 0.001

**Mechanical ventilation (%)**	21 (84.0)	9 (45.0)	0.01

**Creatinine (μmol/L) (mean [SD])**	148 (59)	176 (108)	0.28

**Renal replacement therapy (%)**	7 (28.0)	5 (25.0)	1.0

**G-CSF (%)**	17 (68.0)	9 (47.4)	0.22

**Corticosteroids (%)**	13 (52.0)	9 (47.4)	1.0

**Withdrawal of support (%)**	16 (64.0)	0 (0)	< 0.001

Abbreviations: d = days; SOFA = sequential organ failure assessment; APACHE = acute physiology and chronic health evaluation; MAP = mean arterial pressure; P/F = PaO_2_/FiO_2_; G-CSF = granulocyte colony-stimulating factor.

## Discussion

We conducted a 6.5-year retrospective matched case-control study to compare the clinical characteristics, course, and outcomes of patients with AML admitted to ICU compared with non-critically ill hospitalized AML controls and non-AML critically ill controls.

Our data have several relevant findings. First, while AML patients represent only a minority of all ICU admissions, we found that life threatening illness prompting ICU admission is not uncommon, occurring in 13% of all patients with a diagnosis of AML. Second, these critically ill AML patients had predominantly septic and/or respiratory-related diagnoses and exhibited a high acuity of illness, with the majority presenting with shock. Moreover, AML patients had greater severity of organ dysfunction. Third, AML patients were less likely to receive mechanical ventilation; yet overall they received high intensity support that included vasoactive therapy in 75.6% and RRT in 26.7%, respectively. This translated into longer, though non-significant, stays in ICU and hospital. Fourth, we also found critically ill AML patients had lower adjusted-survival when compared with controls. By univariate analysis, several factors were associated with a lower probability of survival, including older age, AML M1 subtype, higher baseline SOFA score, presence of shock, vasoactive therapy and mechanical ventilation. We also found that worsening SOFA score early after ICU admission correlated with lower survival. Alternatively, receipt of AML-specific chemotherapy while in ICU correlated with higher survival. Finally, changes in patient resuscitation status (i.e. to NFR) and withdrawal of support were both significantly associated with higher likelihood of death at 90-days.

There is a paucity of epidemiological data pertaining to patients with AML requiring life-sustaining measures in the ICU. (Additional File 1) While numerous small series have reported on the clinical outcomes for patients with all forms of hematologic malignancy admitted to ICU, very few have focused on AML, and fewer still have provided estimates of incidence[13,14,16,17]. During a 5-year surveillance, Tremblay et al reported that among 163 consecutive hospitalized AML patients, with a range in AML status (including 38 having received a HSCT), only 32 were admitted to ICU and supported with mechanical ventilation (cumulative incidence 19.6%)[17]. We observed a lower cumulative incidence, with just over 1 in 10 of all newly diagnosed AML patients being supported in ICU; moreover, we observed that AML patients comprised <1% of all ICU admissions during the study period. In a 4-year retrospective study, Merz et al reported on 101 ICU admissions (n = 84) with hematologic malignancies, of which 54.4% had a diagnosis of AML[11]. These AML admissions represented approximately 1.4% of all "emergent and medical" ICU admissions.

Among AML patients admitted to ICU, the in-hospital, 90-day and long-term adjusted-survival were observed to be lower when compared with either hospitalized AML or ICU controls. To our knowledge, this is the first study to evaluate the long-term survival experience of critically ill AML patients compared with matched controls. When compared with an unmatched convenience sample of non-AML critically ill patients, Tremblay et al found AML patients requiring mechanical ventilation to clearly have higher in-hospital mortality[17]. Merz et al found in-hospital mortality noticeably higher for ICU admissions associated with hematologic malignancies compared with unmatched controls (33.7% vs. 10.7%, p < 0.0001)[11]. In general, the observed survival in our study is largely consistent with prior studies, where estimates of ICU, in-hospital and 1-year survival were 12-70%, 3-64%, and 0-34%, respectively [12-14,16,17,20,21,26-34]. The median survival of critically ill AML patients in our cohort was only about three months, in contrast to approximately nine months for the control groups. While the majority of deaths in AML patients occurred early after ICU admission (median duration 5 days), the adjusted risk of death remained significantly higher during follow-up compared with either control group. These data would suggest, in general, that AML patients developing an episode of critical illness prompting ICU support have a less favorable outcome compared with hospitalized AML and non-AML ICU patients.

Prior data imply AML patients supported in ICU have greater health resource utilization. This was shown by Merz et al, who described considerably higher ICU resource use by hemato-oncological patients, as measured by the Therapeutic Interventions Scoring System (TISS) (214 vs. 95, p < 0.0001) and ICU length of stay (2.0 vs. 1.1 days, p < 0.02), compared with emergent medical ICU patients[11]. Despite no difference in the rates of mechanical ventilation or RRT between groups, both of these interventions showed significant correlation with total ICU resource use. This also translated into greater total direct costs per ICU admission for hemato-oncological patients. While our study did not incorporate a formal cost analysis, AML patients in our study had non-significant, but longer observed stays in both ICU and hospital. Interestingly, our data found significantly fewer AML patients received mechanical ventilation, despite evidence of similar or worst lung injury. This observation may, in part, be attributable to prior data correlating less favorable prognosis for these patients requiring mechanical ventilation[17,22,28,35]. These data also suggest greater health resource use for AML patients; however, there is little data on whether this is balanced by higher quality-adjusted survival of ICU admissions with hematologic malignancy. In a small series of 92 critically ill patients with hematologic malignancy, Yau et al described the quality-of-life as acceptable or good in the 7 survivors (7.6%) at 1-year, with no patient reporting significant limitations to daily activities[34].

Few data have explored the long-term disposition of AML patients after life-threatening critical illness. Interestingly, despite lower overall survival, 46.7% of all AML patients supported in ICU in our study survived hospitalization to be discharged home (or 84.0% of survivors). This was statistically comparable to hospitalized AML and ICU controls.

The characterization of potential factors predictive of survival for hematologic malignancy patients prior to and/or early after ICU admission has been the focus of considerable research effort[12,13,16,20,22,26,28,30-32,35]. Several factors have been suggested to predict non-survival at various time points across a range of studies including: older patient age[14], poor performance status[13], AML subtype[13], relapsed/refractory AML status[22], poor cytogenetic profile[16], higher illness severity or organ failure score[13,16,18,22,31,32], leukopenia[12], vasopressor therapy[12,16,22], mechanical ventilation[13,16,22], acute kidney injury[12], absence of bloodstream infection[12,27], and fungal sepsis[32]. In our cohort, we found that older age, AML M1 subtype, higher organ failure score, the presence of shock, vasopressors therapy, and mechanical ventilation were all associated with lower survival. There was no association between survival and cytogenetic profile, therapeutic regimen or burden of co-morbid illness. However, these prognostic factors have been inconsistent and not readily reproducible, due largely to issues related to study design (i.e. small, single centre, retrospective, no controls, analysis) and lack of generalizability (i.e. selection bias, mixed hematologic populations). Of note, we also found receipt of active intensive chemotherapy while in ICU was associated with higher observed survival. Recently, Vandijck et al reported that active chemotherapy was associated with better ICU survival in a cohort of 77 patients admitted to ICU with severe sepsis or septic shock[18]. These observations would suggest that prognosis for AML patients receiving active chemotherapy may be better than perceived.

Perhaps no constellation of disease or treatment-specific factors will consistently predict outcome for all AML patients developing life-threatening critical illness. As such, it may be that prognostic factors may have a limited role in the decision to admit an AML patient to ICU. Indeed, hospital survival in our study was 55.6%, with 84.0% of survivors returning home and 29.9% alive at 1-year. Instead, prognostic factors may have greater relevance for decisions regarding the withholding of life-sustaining therapies and in end-of-life care. For example, worsening illness severity and/or organ dysfunction early after ICU admission has been found to predict poor clinical outcome[11,31,32]. Massion et al showed an improvement in Multi-Organ Dysfunction Score (MODS) of ≤1 point between ICU admission and day 5 correlated with increased mortality (81% vs. 29%, p = 0.001)[32]. Merz et al described significant higher 28-day mortality for patients with worsening SOFA score over the first 48 hours (OR 24.4, 95% CI, 8.8-67.9, p < 0.0001)[11]. Finally, Lamia et al showed worsening Simplified Acute Physiology Score (SAPS) II score, Logistic Organ Dysfunction score (LODS), and SOFA score during the first three days after ICU admission to be the best predictors of in-hospital mortality[31]. Our study further confirmed these observations. We found that the absence of change or an increase in organ failure score early after ICU admission correlated with lower survival.

Furthermore, we found that a change in patient resuscitation status from full resuscitation to not for resuscitation (NFR) and withdrawal of active support were common and both significantly modified the observed mortality at 90-days. In a series of 124 consecutive critically ill hematological malignancy patients, Benoit et al found 4% had a NFR order written within 24 hours of ICU admission, while 20.2% had a NFR order written after a median 7 days in ICU[12]. In our study, only 6.7% of AML ICU patients had a NFR order at presentation, whereas an additional 46.7% had their status later changed to NFR while in ICU. While we did not specifically examine the timing of this change, decisions about withholding life-sustaining measures in our study may have related to higher or worsening organ failure score and need for escalation in treatment intensity (i.e. vasoactive therapy, mechanical ventilation, RRT). We did not find association between change in resuscitation status and age, co-morbidity score, AML subtype, poor AML cytogenetic prognosis, or duration of ICU stay. We recognize one plausible explanation for this finding may relate to a clinician perception of poor clinical outcome and/or general reluctance to pursue aggressive and/or prolonged life sustaining measures for AML patients[36].

There are important limitations to our study. First, our study was single-centered and retrospective in design and was therefore potentially susceptible to bias. For example, we were unable to determine whether AML patients designated as "non-ICU" had end-of-life discussions that precluded admission to ICU, despite comparable "critical illness", which would unduly influence our incidence estimate (i.e. selection bias). However, we have attempted to minimize this by showing no significant differences in the therapeutic regiments received by AML cases and controls. Second, despite over 6 years of surveillance for AML patients admitted to our ICU, our sample was small and provided limited statistical power; we therefore omitted multi-variable analysis for determination of predictors of survival. To compensate, we further matched AML patients based on age, sex and APACHE II score (ICU only). While we did not *a priori *match AML groups for cytogenetic profile or therapeutic regimen received, we found no statistical differences between the AML cases and controls. Third, we endeavored to match the final cohort of AML cases with non-AML ICU controls by a ratio of 1:5; however, we recognize these ICU controls still represent a heterogeneous population and, despite similar illness severity, have observed differences that may impact outcome (i.e. primary diagnosis, prevalence of co-morbid illness). Finally, we were not able to ascertain additional secondary outcomes of relevance to survivors, such as health-related quality of life or functional status and the details of ongoing care requirements, which may also have prognostic significance[34].

## Conclusions

In summary, AML patients may represent a minority of all critically ill admissions; however, are not uncommonly supported in ICU. These AML patients are characterized by high illness severity, multi-organ dysfunction, and high treatment intensity and have a higher risk of death when compared with matched hospitalized AML or non-AML ICU controls. Critically ill AML patients also have greater long-term mortality, which may be associated with severe clinical and disease-specific factors including absence of improvement in organ dysfunction early after ICU admission. Despite lower survival; however, our data imply a significant proportion of these critically ill AML patients survive hospitalization and are discharged home. We contend that additional multi-centre prospective studies are needed to further characterize these outcomes in AML patients suffering an episode of life-threatening critical illness.

## Key messages

• Approximately 1 in 10 patients newly diagnosed with AML develop life-threatening critical illness prompting life-sustaining therapy in ICU.

• AML patients admitted to ICU have greater organ dysfunction, higher treatment intensity and use more health resources compared with matched controls.

• AML patients admitted to ICU have higher short- and long-term mortality compared with matched controls.

• Early absence of change or an increase in organ failure score following admission to ICU was associated with lower likelihood of survival for AML patients.

• Despite the higher mortality observed for AML patients admitted to ICU compared with matched controls, a significant proportion of them survive and are discharged home.

## Abbreviations

AML: acute myelogenous leukemia; APACHE: acute physiology and chronic health evaluation; BMT: bone marrow transplantation; DNR: do not resuscitate; ICU: intensive care unit; LODS: Logistic Organ Dysfunction score; RRT: renal replacement therapy; SOFA: sequential organ failure assessment

## Competing interests

The authors declare that they have no competing interests.

## Authors' contributions

All authors read and approved the final manuscript. ALR and KC developed study protocol, obtained data, analyzed data and wrote manuscript. DRT developed study protocol, and provided critical revision of manuscript. IM developed study protocol, obtained data, analyzed data and provided critical revision of manuscript. SMB conceived the study, developed study protocol, obtained data, analyzed data and wrote and provided critical revision of manuscript.

## Pre-publication history

The pre-publication history for this paper can be accessed here:

http://www.biomedcentral.com/1471-2407/10/516/prepub

## Supplementary Material

Additional file 1 **Summary of clinical studies evaluating clinical outcomes in critically ill patients with hematologic malignancies**. Click here for file
