# Supplementary material for: Clinical characteristics and outcomes of patients with acute myelogenous leukemia admitted to intensive care: a case-control study
Source: BMC Cancer. 2010 Sep 28;10:516. doi: 10.1186/1471-2407-10-516 (PMC2955611; doi:10.1186/1471-2407-10-516)
Supplement: Additional file 1 — Summary of clinical studies evaluating clinical outcomes in critically ill patients with hematologic malignancies. [file 1471-2407-10-516-S1.DOC]

**Additional File 1: Summary of clinical studies evaluating clinical outcomes in critically ill patients with hematologic malignancies.**

| **Study** | **Year** | **Design** | **N** | **Population** | **Control Group** | **Outcomes** | **Prognostic factors** |
| --- | --- | --- | --- | --- | --- | --- | --- |
| **Schuster et al[8]** | 1983 | Retrospective | 77 | HM | No | MR: ICU 60%; Hospital 80% | Hospital mortality^1^ – ICU LOS>2 wks, MV>5 d |
| **Estopa et al[5]** | 1984 | Retrospective | 30 | HM + respiratory failure | No | MR: ICU 80%; Hospital 93%; 2-yr 97% | Not evaluated |
| **Peters et al[21]** | 1988 | Retrospective | 119 | HM + MV | No | MR: Hospital 82%  Median survival from hosp discharge 1-yr | Hospital mortality^1^ – acute leukemia, NHL |
| **Johnson et al[6]** | 1986 | Retrospective | 26 | HM | Unmatched  (HM ward, n=120) | MR: Hospital 69% (ICU) vs. 16% (ward)  ICU LOS: Survivors 3.2 d vs. dead 9.4 d | Hospital mortality^1^ – ↑APACHE II, ↑age, respiratory failure |
| **Ashkenazi et al[9]** | 1986 | Retrospective | 29 | Acute leukemia (n=21)/acute lymphoma (n=8) | No | MR: Hospital 69% | ICU mortality^1^ – ↑age, later stage disease, ↑APS, MV |
| **Lloyd-Thomas et al[7]** | 1986 | Retrospective | 22 | HM | No | MR: ICU 55%; Hospital 82% | ICU mortality^1^ – APS >15  Hospital mortality^1^ – ↑organ failure, no response to chemo, poor recovery of BM post-chemo |
| **Lloyd-Thomas et al[20]** | 1988 | Retrospective | 60 | HM | No | MR: ICU 63%; Hospital 78% | Hospital mortality^1^ – ↑APACHE II; ↑ organ failure, no BM recovery post-chemo, relapse, leucopenia |
| **Brunet et al[10]** | 1990 | Retrospective | 260 | HM | No | MR: ICU 43%; Hospital 57% | ICU mortality^2^ – ↑SAPS, combination MV + RRT, septic shock, ICU LOS |
| **Yau et al[34]** | 1991 | Retrospective | 92 | HM | No | MR: ICU 65%; Hospital 77%; 1-yr 86%; 3-yr 90%  Median survival post-discharge 23 mo  3-yr QOL good for survivors (n=7) | Not evaluated |
| **Tremblay et al[17]** | 1995 | Retrospective | 32 | AML/BMT + MV | Unmatched | MR: ICU 88%; Hospital 97%; 4 mo 100% | ICU mortality^1^ – ↑APACHE II |
| **Epner et al[19]** | 1996 | Retrospective | 157 | HM + MV | No | MR: Hospital 83% | Hospital mortality^2^ – ↑age, ↑APACHE III, BMT, relapse, neutropenia>30 d |
| **Ewig et al[29]** | 1998 | Retrospective | 89 | HM | No | MR: ICU 79% | ICU mortality^2^ – MV, BMT |
| **Evison et al[28]** | 2001 | Retrospective | 78 | HM | No | MR: ICU 26%; 60 day 41% | ICU mortality^2^ – ↑organ failure score  60 d mortality^2^ – ↑organ failure score, ↑liver enzymes |
| **Rabbat et al[13]** | 2005 | Retrospective | 83 | AML | No | MR: ICU 34%; 12 mo 66% | ICU mortality^1^ – ↑SAPSII, IMV (vs NIMV), ↑organ failure score  12 mo mortality^2^ – no remission |
| **Massion et al[32]** | 2002 | Retrospective | 84 | HM | No | MR: ICU 38%; Hospital 61%; 6 mo 75% | ICU mortality^2^ – MV, fungal infection  Hospital mortality^2^ – fungal infection, BMT, ↑MODS, no improved ∆MODS  6 mo mortality^2^ – BMT, tumor progression, AML, NHL |
| **Krochinsky et al[30]** | 2002 | Retrospective | 104 | HM | No | MR: ICU 44%; 6 mo 67%; 1-yr 71% | ICU mortality^2^ – MV, ↑SAPS II, ↑CRP  6 mo mortality^2^ – MV |
| **Benoit et al[12]** | 2003 | Retrospective | 124 | HM | No | MR: ICU 42%; Hospital 54%;6 mo 67% | Hospital mortality^2^ – leukopenia, vasopressors, urea>12 mmol/L; blood-stream infection |
| **Silfvast et al[33]** | 2003 | Retrospective | 30 | HM | No | MR: ICU 43%; 3 mo 77%; 1-yr 80% | Hospital mortality^2^ – day 1 SOFA >11, increasing SOFA  1-yr mortality^2^ – disease status |
| **Depuydt et al[27]** | 2004 | Retrospective | 166 | HM + MV | No | MR: ICU 62%; Hospital 71% | Hospital mortality^2^ – male, ↑SAPS II, intubation >24 hr, AML diagnosis |
| **Rabe et al[14]** | 2004 | Retrospective | 30 | AML + MV | No | MR: ICU 87%  Median survival from post-discharge 303 d | ICU mortality^1^ – age≥50y, APACHE II>26, refractory septic shock, transformed AML |
| **Cornet et al[26]** | 2005 | Retrospective | 58 | HM | No | MR: ICU 62%; 1-yr 88% (overall); 1-yr 67% (discharged); 3-yr 95%  Median survival post-discharge 307 d | ICU mortality^1^ – ↑SOFA, ↓platelets, ↑INR, ↑bilirubin,  1-yr mortality^1^ – ∆SOFA (no improvement) |
| **Lamia et al[31]** | 2006 | Retrospective | 92 | HM | No | MR: ICU 50%; Hospital 58% | Hospital mortality^2^ – ↑SAPSII, ↑organ failure scores, ∆organ failure scores (no improvement) |
| **Cherif et al[35]** | 2007 | Retrospective | 85 | HM (88%) | No | MR: ICU 30%; 30 d 49%; 6 mo 62%;  5-yr 80% | ICU mortality^2^ – ↑APACHE II, MV  30 d mortality^2^ – MV  6 mo mortality^2^ – malignancy |
| **Merz et al[11]** | 2008 | Retrospective | 101* | HM | Unmatched  (ICU, n=3808) | MR (HM): Hospital 34%; 28 d 30%;  90 d 40%  MR (ICU controls): Hospital 11% | ICU mortality – ↑SAPSII, MV, RRT  Hospital mortality – ↑SAPS II  28 d mortality – ↑48 hr SOFA, ↑SAPS II |
| **Thakkar et al[16]** | 2008 | Retrospective | 85 | AML/ALL | Matched  (ward, n=243) | MR (cases): ICU 68%; Hospital 73%; 6 mo 82%; 12 mo 84%  Median survival: AML 199 d, ALL 192 d  MR (ward controls): 2 mo 15%; 6 mo 31%; 12 mo MR 54% | ICU/2 mo mortality^2^ – BMT chemo, ΔAPACHE II (worse)  6 mo mortality^2^ – vasopressors, cytogenetics  12 mo mortality^2^ – ΔAPACHE II (worse) |
| **Park et al[22]** | 2008 | Retrospective | 50 | All forms of acute leukemia | No | MR: ICU 60%; Hospital 68%; 12 mo 70% | ICU mortality^2^ – refractory/relapse status, ↑ admission SOFA score |

Abbreviations: AML = acute myelogenous leukemia, ALL = acute lymphoblastic leukemia, HM = hematologic malignancy (include acute leukemias, chronic leukemias and all forms of lymphomas), NHL = Non-Hodgkin’s lymphoma, MR = mortality rate, MV = mechanical ventilation, IMV = invasive mechanical ventilation, NIMV = non-invasive mechanical ventilation, RRT = renal replacement therapy, BMT = bone marrow transplant, chemo = chemotherapy, AT-III = antithrombin III, CRP = C-reactive protein, APS = acute physiology score, ICU = intensive care unit, QOL = quality of life, d = day, mo = month, yr = year; Δ = delta or change in.

^1^ = Univariate analysis, ^2^ = Multivariate analysis

*Represents consecutive admissions for 84 patients
